# Maternal and fetal mitochondrial gene dysregulation in hypertensive disorders of pregnancy

**DOI:** 10.1152/physiolgenomics.00005.2023

**Published:** 2023-05-15

**Authors:** Contessa A. Ricci, Danielle M. Reid, Jie Sun, Donna A. Santillan, Mark K. Santillan, Nicole R. Phillips, Styliani Goulopoulou

**Affiliations:** ^1^Department of Physiology and Anatomy, University of North Texas Health Science Center, Fort Worth, Texas, United States; ^2^Department of Microbiology, Immunology and Genetics, University of North Texas Health Science Center, Fort Worth, Texas, United States; ^3^Department of Obstetrics and Gynecology, University of Iowa Carver College of Medicine, Iowa City, Iowa, United States; ^4^Department of Gynecology and Obstetrics, Lawrence D. Longo MD Center for Perinatal Biology, Loma Linda University School of Medicine, Loma Linda, California, United States

**Keywords:** hypertension, mitochondrial DNA, placenta, preeclampsia

## Abstract

Mitochondrial dysfunction has been implicated in pregnancy-induced hypertension (PIH). The role of mitochondrial gene dysregulation in PIH, and consequences for maternal-fetal interactions, remain elusive. Here, we investigated mitochondrial gene expression and dysregulation in maternal and placental tissues from pregnancies with and without PIH; further, we measured circulating mitochondrial DNA (mtDNA) mutational load, an index of mtDNA integrity. Differential gene expression analysis followed by Time Course Gene Set Analysis (TcGSA) was conducted on publicly available high throughput sequencing transcriptomic data sets. Mutational load analysis was carried out on peripheral mononuclear blood cells from healthy pregnant individuals and individuals with preeclampsia. Thirty mitochondrial differentially expressed genes (mtDEGs) were detected in the maternal cell-free circulating transcriptome, whereas nine were detected in placental transcriptome from pregnancies with PIH. In PIH pregnancies, maternal mitochondrial dysregulation was associated with pathways involved in inflammation, cell death/survival, and placental development, whereas fetal mitochondrial dysregulation was associated with increased production of extracellular vesicles (EVs) at term. Mothers with preeclampsia did not exhibit a significantly different degree of mtDNA mutational load. Our findings support the involvement of maternal mitochondrial dysregulation in the pathophysiology of PIH and suggest that mitochondria may mediate maternal-fetal interactions during healthy pregnancy.

**NEW & NOTEWORTHY** This study identifies aberrant maternal and fetal expression of mitochondrial genes in pregnancies with gestational hypertension and preeclampsia. Mitochondrial gene dysregulation may be a common etiological factor contributing to the development of de novo hypertension in pregnancy-associated hypertensive disorders.

## Graphical abstract

**Figure:**
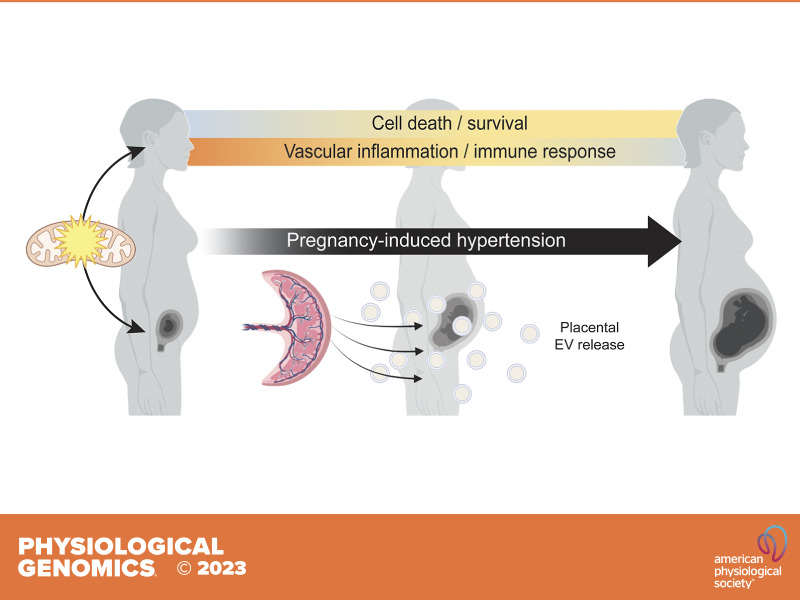

## INTRODUCTION

Hypertensive disorders of pregnancy, including gestational hypertension and acute hypertensive syndromes such as preeclampsia, are one of the leading causes of maternal and fetal mortality and morbidity worldwide (1, 2). Gestational hypertension is the presence of de novo hypertension in a previously normotensive woman, whereas preeclampsia is a heterogenous and complex syndrome diagnosed as new-onset hypertension after 20 wk of gestation with proteinuria and/or end-organ damage (3). The mechanisms underlying the clinical features of these pregnancy-induced hypertensive disorders (PIH) remain unclear, despite intensive research efforts.

There is growing evidence that dysregulated or impaired mitochondrial function may play a role in PIH. The involvement of mitochondrial dysfunction in preeclampsia was first reported in 1989, when Torbergsen et al. (4) described a family with mitochondrial dysregulation coinciding with a high incidence of preeclampsia and eclampsia, which is a severe complication of preeclampsia. Since then, various scientific reports have provided evidence of impaired mitochondrial fusion/fission dynamics (5, 6), lower activity of complex II of the electron transport chain, reduced expression of complexes I and IV, and mitochondrial swelling and broken cristae in placentas from pregnancies with preeclampsia compared with placentas from healthy pregnancies (7). Furthermore, we have reported that pregnant patients with preeclampsia have reduced concentrations of circulating cell-free mitochondrial DNA (mtDNA) compared with healthy normotensive pregnant individuals, with more mtDNA transported in vesicular structures compared with membrane-free form (8). These findings may be highly relevant to PIH pathophysiology, as circulating cell-free mtDNA is often used as an accessible blood-based surrogate of mitochondrial function, cellular stress, and systemic inflammation (9–12).

Although the number of studies concerning the involvement of mitochondrial dysfunction in PIH is growing, the role of mitochondrial gene regulation is still incompletely understood. In addition, maternal- versus fetal-mediated mitochondrial contributions to PIH pathophysiology have not been delineated. Thus, the main objective of this study was to determine the role of mitochondrial gene dysregulation in pregnancies with de novo hypertension, namely preeclampsia and gestational hypertension. We used publicly available high-throughput transcriptomic data sets that had been sequenced from maternal peripheral blood plasma and placentas from normotensive pregnancies and PIH cases. These data sets were chosen because the maternal circulating cell-free transcriptome (13) can provide a systemic view of primarily maternal mitochondrial processes (with modest placental contributions; 14), whereas the placental transcriptome allows examination of fetal-specific mitochondria-mediated processes.

Transcriptomic data used here focused specifically on cases of de novo hypertension (gestational hypertension and preeclampsia) to gain insight into mitochondrial commonalities related to hypertension (vs. disease progression or severity). We hypothesized that *1*) evidence of mitochondrial dysregulation, indicated by aberrant expression of mitochondrial genes, would be present in maternal and fetal tissues from patients with PIH; *2*) putative consequences of mitochondrial dysregulation in PIH, indicated by expression patterns of genes that are co-dysregulated with mitochondrial genes, would mirror processes known to be involved in PIH pathology. We also performed mitochondrial DNA (mtDNA) mutational load analysis in maternal peripheral blood mononuclear cells (PBMCs) from a separate cohort of patients with preeclampsia and healthy controls. We hypothesized that the mitochondrial genome mutational load in PBMCs from pregnancies with PIH would be higher compared with normotensive pregnancies.

## MATERIALS AND METHODS

### Data Set and Subject Characteristics

To assess mitochondrial dysregulation in PIH, we reanalyzed publicly available data deposited to NCBI’s Gene Expression Omnibus (15), a database of preprocessed gene expression data sets. After examination of >200 entries generated using the search criteria “preeclampsia,” two high-throughput sequencing transcriptomic data sets were chosen based on: *1*) being a longitudinal, prospective maternal study and a placental study sequenced on comparable platforms, *2*) being conducted in humans and not using cell culture, *3*) having an accompanying peer-reviewed publication to validate methods against, 4) being of sufficient sample size.

Our first transcriptomic data set is from maternal peripheral venous blood plasma of nine normotensive pregnancies and eight cases of PIH [preeclampsia (*n* = 5); gestational hypertension (*n* = 3); GEO accession GSE154377 (16)] were used to investigate maternal mitochondrial gene expression. We acknowledge that a small portion of this expression may be placenta-derived but refer to this as a maternal because we make the assumption that this is the predominant signal. Patient-specific data can be found in the original publication (16). These data were sequenced on the Illumina HiSeq 4000 platform. Data were collected at four time points *1*) at the end of the first trimester (1st trimester, 12–17 wk of gestation), *2*) at the end of the 2nd trimester (2nd trimester, 18–22 wk of gestation), *3*) at the end of the 3rd trimester (3rd trimester, 35–37 wk of gestation), and *4*) at delivery (time of collection unspecified in original publication). The second data set included data from placental samples collected within 30 min of delivery from 21 normotensive pregnancies and 20 pregnancies with preeclampsia [GEO accession GSE114691 (17)]. Placenta samples were collected from two central and two peripheral locations, pooled, and sequenced on the Illumina HiSeq 2000 platform. The maternal decidua was removed from all samples before analysis. Therefore, we refer to these data as data from fetal samples. Patient-specific data can be found in the original publication (17). For both studies, detailed descriptions of the informed consent process and diagnosis criteria of preeclampsia and gestational hypertension can be found in the original papers (16, 17).

For the mtDNA mutational load analysis, deidentified subject information, and PBMC samples were acquired from the Maternal Fetal Tissue Bank (MFTB, IRB No. 200910784) of the Women’s Health Tissue Repository at the University of Iowa Hospitals and Clinics. Samples of maternal PBMCs from 10 pregnancies with preeclampsia and 13 normotensive pregnancies were analyzed. Samples from cases and healthy pregnant controls were matched for gestational age at sampling. Characteristics of subjects are listed in Supplemental Table S1; all Supplemental Tables are available at https://doi.org/10.6084/m9.figshare.21970694, and sample collection methods are detailed in Ref. 8.

### Detection of Differentially Expressed Mitochondrial Genes and Interaction Genes

To identify differentially expressed mitochondrial genes (mtDEGs) present in maternal peripheral blood plasma samples, differential gene expression analysis was conducted using the DESeq2 package (18) available for R software (19). Patient outliers were identified and removed using median absolute distance of whole gene expression profile. Final data set is composed of eight normotensive and eight PIH cases. After outlier removal, contrasts were conducted for each collection timepoint (e.g., one contrast between normotensive and PIH pregnancies for 1st trimester, one contrast between normotensive and PIH for 2nd trimester, etc.).

To build DESeq2 contrast models for maternal data, patient covariates were first investigated for autocorrelation, which found neonatal birth weight to be correlated with placental weight, neonatal head circumference, and length (Supplemental Fig. S1; all Supplemental Figures are available at https://doi.org/10.6084/m9.figshare.21970706). Maternal age and body mass index (BMI) were separately correlated (Supplemental Fig. S1). Correlated fetoplacental biometrics (placental weight, infant weight, and infant length) were collapsed into a summary statistic (utero summary statistic) by conducting principal component analysis (PCA) and taking the eigenvector associated for PC1 (>60% variance explained, data not shown) for each patient. This process was also carried out to collapse maternal BMI and maternal age into an additional summary statistic (Age-BMI summary statistic) for each patient (>60% variance explained, data not shown). Patient covariates significantly stratifying gene expression were then determined by conducting PCA on the raw transcript counts for each timepoint, followed by general linear model testing for covariate effects (main effects and interactive effects with PIH diagnosis) on PC1 loading values. Covariates meeting *P* values ≤ 0.1 were included in DESEq2 contrast models to allow for greater sensitivity in DESeq2 models (Supplemental Table S2). After conducting each contrast, DEGs were considered genes meeting a *P*-adjusted value ≤ 0.05 and an absolute log2 fold change ≥ 0.5 (Supplemental File S1; see https://doi.org/10.6084/m9.figshare.21970595).

An in-house DESeq analysis could not be conducted for fetal samples as individual patient and neonatal characteristics did not accompany sequencing data in the original publication. We, therefore, relied on the DEG list provided by the authors [Supplemental material of original publication (17)] to identify fetal DEGs. In fetal data, only adjusted *P* values of significance and direction of effect were provided. Therefore, to understand fetal mtDEG dynamics, the adjusted *P* values for DEG list genes identified as mtDEGs were converted to Z-scores using one-tailed standard *P* value distributions. We reported the lower tail (negative values) for downregulated gene expression and the upper tail (positive values) for upregulated gene expression. Standard error was not provided by authors.

To identify mtDEGs, DEGs in both maternal and fetal data were matched to the MitoCarta 3.0 database (20), a database of all genes encoded by both the mitochondrial and nuclear genome whose protein products have verified residence within the mitochondria organelle (Supplemental Table S3). After mtDEGs were identified, respective mtDEG interaction genes expressed in both maternal and fetal data were compiled. mtDEGs interaction genes were defined as genes meeting an interaction confidence of “medium” or better (interaction score ≥ 0.400) using String databases (21). For maternal longitudinal analyses throughout pregnancy (1st trimester, 2nd trimester, and 3rd trimester), all interaction genes expressed were used to compile relevant files. For both maternal and fetal functional enrichment analyses at delivery, respective mtDEG interaction genes were identified in DEGs detected at delivery (details in *Functional Enrichment Analyses*). Supplemental Fig. S2 (available at https://doi.org/10.6084/m9.figshare.21970706) provides a diagrammatic representation of the analytical process just described.

### Maternal Longitudinal Analysis of Mitochondrial Gene Expression and mtDEG Interaction Gene Expression during Gestation

To determine expression patterns of maternal mtDEGs and respective interaction genes throughout pregnancy, Time Course Gene Set Analysis (TcGSA) was conducted using the TcGSA package (package version 0.12.6) available for R (22). Default settings for TcGSA models were used. Delivery collection data were excluded from this analysis due to the unique physiology of labor and delivery.

Raw transcript counts were total count normalized (23) by counts per million. A custom gene matrix transposed (GMT) file (Supplemental File S2; see https://doi.org/10.6084/m9.figshare.21970655) was made using the interaction genes for each mtDEG identified during gestation, where gene sets were composed of the interaction genes identified using String databases for each respective mtDEG (described earlier). A null TcGSA model to test if significant gene sets detected were artifacts of gene set size was conducted by running a second TcGSA model with a separate, size-matched GMT file composed of nonmitochondrial and nondifferentially expressed genes. Significant gene sets were considered those meeting an adjusted *P* value ≤ 0.05 after TcGSA. Significant gene sets were then used in functional enrichment analyses (details in *Functional Enrichment Analyses*). Supplemental Fig. S3 provides a diagrammatic representation of the maternal longitudinal analysis just described.

### Functional Enrichment Analyses

Functional enrichments for mtDEG interaction genes present in maternal and fetal tissues were conducted using Gene Ontology enrichments [GO enrichment analysis (24)] and/or Ingenuity Pathway Analysis (IPA, QIAGEN Inc., https://digitalinsights.qiagen.com/IPA). For maternal longitudinal analysis during pregnancy, GO enrichment analyses were first conducted on individual significant gene sets detected by TcGSA using the Fisher’s exact test on the Gene Ontology web server and false discovery rate correction. This preliminary probe was done before IPA because *1*) mtDEG interaction genes primarily involve mitochondrial organelle homeostasis and metabolic homeostasis, which obscured less prominent signals of biological and pathological importance; and *2*) IPA could only be carried out by combining gene sets due to small sizes of individual gene sets. Full GO term lists for each gene set can be found in Supplemental File S2. Two of the five significant gene sets detected by TcGSA, *MRPL38* and *FKBP8* gene sets, included enrichments for terms involved in immune system processes, developmental processes, and apoptotic processes (among others; Supplemental File S3; see https://doi.org/10.6084/m9.figshare.21970664) and were therefore combined for IPA at each gestational observation (1st trimester, 2nd trimester, and 3rd trimester) to determine pathway enrichment changes through time. IPA was conducted using the Ingenuity Knowledge Base genes only reference set and specifying Human as the sample species. All other parameters followed default settings (refer to Supplemental Fig. S3).

Due to the limited number of interaction genes present during delivery for both maternal and fetal tissues, IPA was not feasible. Therefore, GO enrichments were used to carry out functional enrichment analyses of mtDEG interaction genes in maternal and fetal tissues at delivery. GO enrichment analyses for biological processes and cellular components were conducted on mtDEG interaction genes (separate analyses for maternal and fetal data) using default settings on the Gene Ontology web server as described earlier. Full terms lists can be found in Supplemental File S3.

For plotting purposes, GO terms were matched to individual mtDEG interaction genes, and corresponding *P*-adjusted values, which are in reference to the normotensive subjects per data set at delivery, for GO term enrichments were converted to Z-scores as described earlier for individual mtDEG interaction genes and using the log-fold change to inform the direction of the Z-score (Supplemental File S4; see https://doi.org/10.6084/m9.figshare.21970667). Terms were then condensed using the “reduce_overlap” function in the GO Plot package (25) available for R, specifying a 99% overlap as the criteria for term collapsing. Supplemental Fig. S4 provides a diagrammatic representation of maternal and fetal functional enrichment analyses at delivery as just described.

### Mutational Load Analysis

Mutational load analysis was carried out on PBMCs using whole mitochondrial genome amplification followed by deep Illumina-based next generation sequencing. Whole mtDNA was amplified via REPLI-g Human Mitochondrial DNA kit (Qiagen, Venlo, Netherlands) following the manufacturer protocol. The method of amplification uses phi29 polymerase-based rolling circle and multiple displacement amplification. This method was selected to effectively enrich for the mitochondrial genome as opposed to nuclear DNA, as a means for sufficient coverage for subsequent whole genome sequencing targeting mtDNA. The sequencing library for each sample was prepared via the Nextera XT DNA Library Preparation kit (Illumina, San Diego, CA) following the manufacturer protocol for multiplexing. The sequencing library was sequenced on the NextSeq 550 Sequencer (Illumina) platform. Raw sequenced reads were aligned to the human mitochondrial reference genome hg38 via BWA-MEM (v0.7.17) using the default parameter for mapping to produce corresponding sequence alignment and map (SAM) files (26). The generated SAM files were processed with SAMtools (v.1.9) to produce binary alignment and map (BAM) files that were sorted, indexed, and statistically assessed by coordinate (27). Reads within the generated files were assigned to a single new read-group through Picard via the AddOrReplaceReadGroups tool (http://broadinstitute.github.io/picard). The resultant single read-group BAM files were further processed to remove duplicate reads using the GATK4 Spark application of the Picard tool MarkDuplicates (28). SAMtools (v.1.9) was used to index the reads followed by somatic variant calling via GATK4 Mutect2 utilizing the mitochondrial mode and excluding read orientation base qualities below 30 (28, 29). Variants confirmed in both read directions were tallied. The data conformed to normality and equal variance (Supplemental Fig. S5), and an unpaired *t*-test (one-sided) was used to test the hypothesis that mothers with preeclampsia have elevated mtDNA mutational loads. *T*-test and graphs were generated using Prism v.9.1.1. Because the American Statistical Association discourages sole reliance on “bright line” rules (e.g., *P* < 0.05) in the interpretation of statistical tests, and instead recommends *P*-value interpretation in the context of effect size estimations (30) [defined as the discrepancy between the null hypothesis and the alternate hypothesis being tested (31)], we additionally calculated the Cohen’s *d* estimation for effect size (31–33) using the “cohen.d” function in the EffSize package (34) available for R. A Cohen’s *d* of ∼0.2 is considered a small effect size, whereas medium effect sizes are ∼0.5, and large-effect sizes are ∼0.8 (33, 34).

## RESULTS

### Mitochondrial Gene Expression through Pregnancy and at Delivery

**Figure 1.**
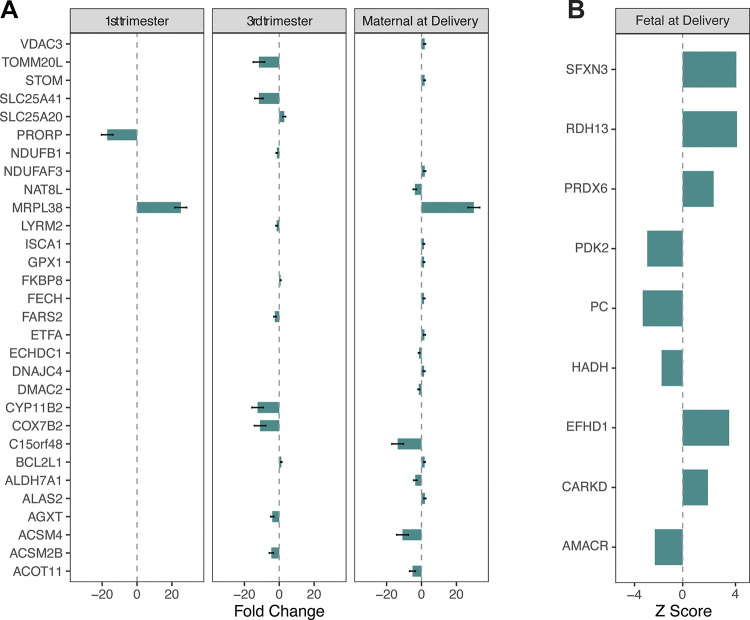
Maternal and fetal mtDEGs detected. *A*: maternal mtDEGs in blood plasma throughout gestation and at delivery (no mtDEGs were detected in 2nd trimester). *B*: fetal mtDEGs in placenta at delivery; *P*-adjusted values of significance and expression directions provided in the original publication were converted to z-scores; error bars not present because standard error was not reported in original publication (17).

Thirty differentially expressed mtDEGs were detected in the maternal cell-free circulating transcriptome (all gestational ages combined; Fig. 1*A*), and nine were detected in placental transcriptome (Fig. 1*B*). Only two mtDEGs were shared between maternal gestational ages: *MRPL38* (1st trimester and at delivery) and *BCL2L1* (3rd trimester and at delivery).

### Maternal Mitochondrial Interaction Gene Expression through Pregnancy

**Figure 2.**
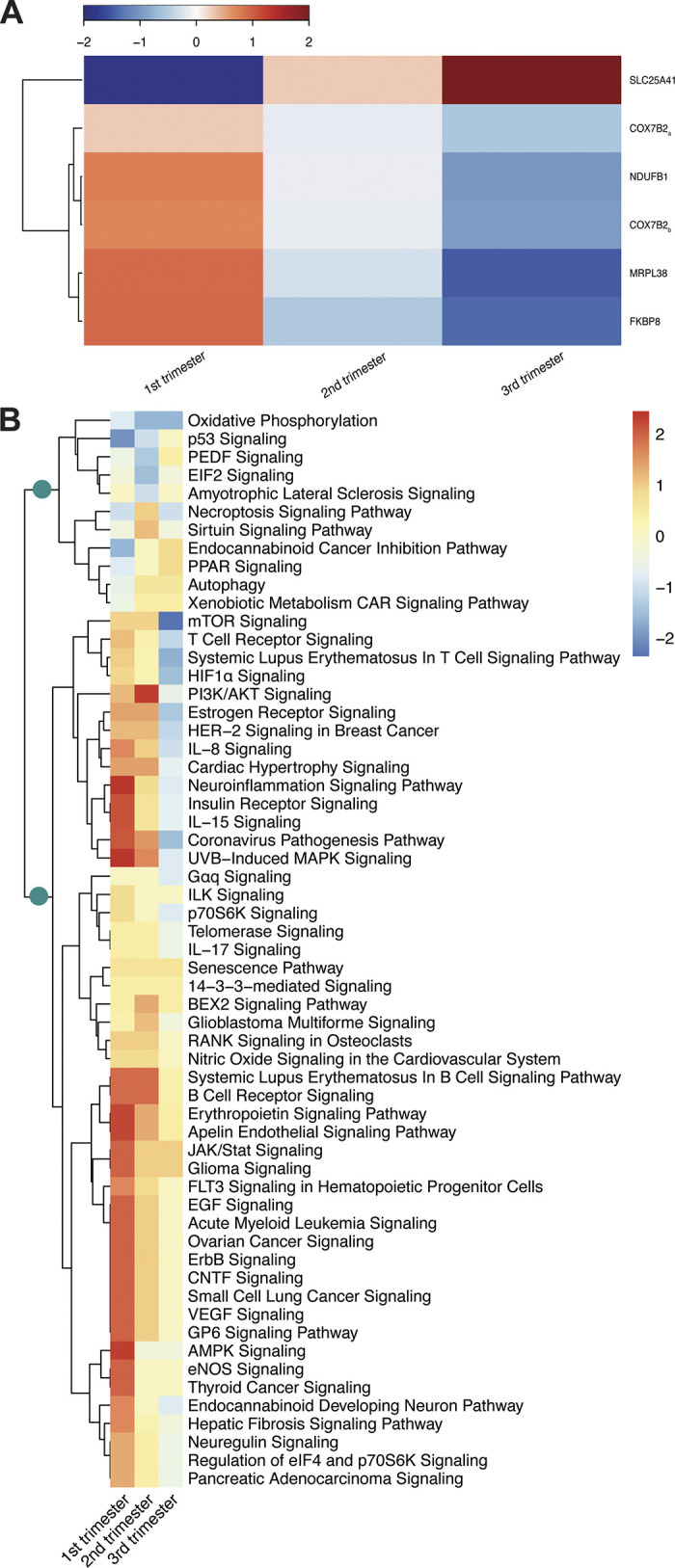
Maternal mitochondrial interaction gene expression through gestation. *A*: mitochondrial interaction gene sets significantly affected by time and gestational age; subscript for COX7B2 indicate two separate but significant gene expression trends were detected. *B*: hierarchical clustering of differentially regulated pathways in *MRPL38* and *FKBP8* interaction genes; teal circles represent groupings of cell death/survival pathways and immune system pathways.

Of the 14 maternal mtDEGs specific to gestation, five displayed changes in the expression of genes within their interaction networks (hereon referred to as mitochondrial interaction genes) that were differentially affected by gestational age and condition (Fig. 2*A*; Supplemental Table S4). Of these, all but 1 (*SLC25A41* interaction genes) displayed decreased expression through gestation in mothers with PIH. All five significant gene sets were primarily enriched for mitochondrial and metabolic homeostatic processes [Gene Ontology enrichment (24); Supplemental File S3].

Pathway analysis (35; Ingenuity Pathway Analysis, QIAGEN Inc., https://digitalinsights.qiagen.com/IPA) of *MRPL38* and *FKBP8* interaction genes in hypertensive mothers found a significant portion of pathways previously implicated in PIH pathophysiology (∼69.5% of 59 total pathways, Supplemental Table S5; Fig. 2*B*). Pathways hierarchically clustered into two main branches: a smaller group overrepresented by cell death/survival pathway (∼45% of 11 total pathways; Supplemental Table S5) displaying downregulation in early pregnancy that transitioned to upregulation in late pregnancy; and a larger group overrepresented by immune system pathways (inflammation in particular; ∼35% of 48 total pathways; Supplemental Table S5) displaying upregulation in early pregnancy that was either attenuated or transitioned to downregulation in late pregnancy. Across both branches, a number of differentially expressed pathways were previously implicated in placental angiogenesis and/or vasculogenesis (∼25% of 107 total pathways, Supplemental Table S5; Fig. 2*B*), with several others having also been implicated in angiogenesis generally (data not shown).

### Mitochondrial Interaction Genes at Delivery

**Figure 3.**
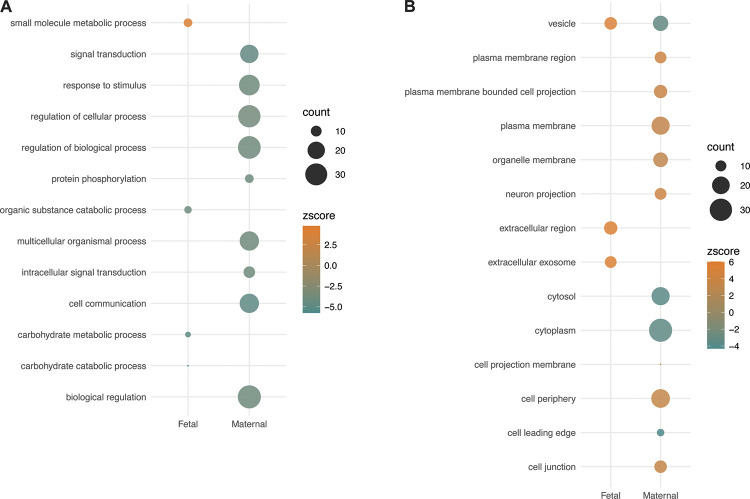
Expression patterns and functional enrichment analysis of maternal and fetal mitochondrial interaction genes at delivery. *A*: biological process enrichments for hypertensive maternal and fetal tissues at delivery. *B*: cellular component enrichments for hypertensive maternal and fetal tissues at delivery. Z-score magnitude and direction are in reference to the normotensive subjects at delivery. Terms condensed for plotting purposes if genes comprising the term shared ≥ 99% overlap. “Count” represents number of genes comprising term.

Eighteen maternal mtDEGs were detected at delivery, but only 37 mitochondrial interaction genes were simultaneously differentially expressed (Supplemental File S5 available at https://doi.org/10.6084/m9.figshare.21970670). Likewise, the 9 mtDEGs detected in placentas were associated with only 13 differentially expressed mitochondrial interaction genes (Supplemental File S5). Maternal mitochondrial interaction genes were enriched [Gene Ontology enrichment (24)] for ∼9 main biological processes in hypertensive mothers (Fig. 3*A*; Supplemental File S5) but lacked a unifying theme. Fetal mitochondrial interaction genes in hypertensive pregnancies were only enriched for metabolic biological processes (Fig. 3*A*; Supplemental File S5).

Fetal mitochondrial interaction genes were enriched for 21 cellular components that were almost exclusively indicative of cellular secretion and extracellular vesicle (EV) release (Fig. 3*B*; Supplemental File S5). Conversely, cellular components differentially enriched among mitochondrial interaction genes in hypertensive mothers also lacked a unifying theme and shared only a single term (vesicles) with fetal enrichments (Fig. 3*B*; Supplemental File S5).

### Maternal Mitochondrial Genome Mutational Load

**Figure 4.**
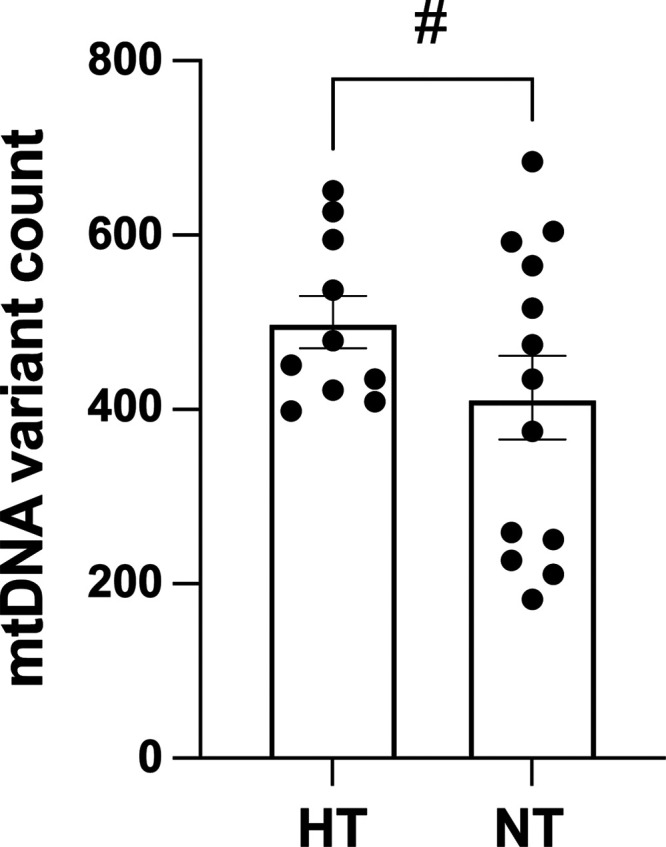
Maternal mitochondrial genome mutational load. “HT”: hypertensive pregnancies (*n* = 10); “NT”: normotensive pregnancies (*n* = 13); “#”: trending on significance (unpaired *t*-test, *P* value = 0.08), indicating unlikely adherence to the null model of no difference; medium effect size observed (Cohen’s *d* = 0.60), indicating biological importance.

Maternal mitochondrial genome mutational loads in mothers with preeclampsia trended toward greater abundance but did not pass statistical significance (*P* value = 0.08, unpaired *t*-test; Cohen’s *d* = 0.06; Fig. 4).

## DISCUSSION

Our main findings show *1*) differential expression of mitochondrial genes (mtDEGs) in maternal and fetal tissues from PIH compared with normotensive pregnancies; *2*) no detected mtDEGs were shared between maternal data sets and fetal data sets, and all detected mtDEGs were nuclear and not mitochondria-encoded; *3*) in PIH, maternal mitochondrial dysregulation was associated with pathways involved in inflammation, cell death/survival, and placental development, whereas fetal mitochondrial dysregulation was associated with increased EV production at term.

Differential expression of mitochondrial genes (mtDEGs), which alludes to dysregulation of mitochondria organellar function (36, 37), was present in PIH maternal (30 mtDEGs) and fetal (9 mtDEGs) tissues. No detected mtDEGs were shared between maternal and fetal data sets, and all detected maternal and fetal mtDEGs were nuclear-encoded. This may be explained by the semiautonomous nature of the mitochondria organelle. The number of mitochondria-encoded genes are limited [Complex I/III/IV/V subunits and rRNAs/tRNAs for mitochondrial translation (38–40)] and are less regulated than nuclear-encoded mitochondrial genes (38, 41, 42). Mitochondrial fidelity is also largely driven by nuclear-encoded mitochondrial genes [e.g., master regulators for mitochondrial biogenesis (39) and dynamics (39, 40)]. Nuclear-encoded mitochondrial genes are implicated in multiple disease pathologies (43–45). Previous work has also demonstrated skeletal muscle (46) defects and cardiac muscle (47) defects in mouse knockouts of the nuclear-encoded mitochondrial gene *Tfam*. This finding implicates nuclear processing of mitochondrial components as an important factor in PIH pathophysiology and may be an additional link between preeclampsia and cardiovascular disease.

The presence of mtDEGs (30 maternal, 9 fetal) from different PIH tissues originating from two independent studies suggests mitochondrial dysregulation is a widespread phenomenon in pregnancies with new-onset hypertension. In addition, processes carried out by mtDEG interaction genes detected in the maternal circulating cell-free transcriptome through gestation were primarily involved in mitochondrial function and homeostatic pathways. Because the maternal circulating cell-free transcriptome describes RNA primarily bound in EVs (13), and because EV cargo reflect the state of originating cells (48, 49), the prioritization of mitochondrial function and homeostasis also suggests systemic maternal mitochondrial dysregulation in PIH. Identifying transcript tissue of origin or specific biological process was beyond the scope of this study, but prior work shows significant contributions to the circulating cell-free transcriptome from leukocytes (48) and the placenta during pregnancy (50), potentially via activation of apoptosis- or necrosis-related mechanisms (51).

Processes associated with five mtDEGs (*MRPL38*, *FKBP8*, *COX7B2*, *SLC25A41*, and *NDUFB1*) in the maternal cell-free circulating transcriptome were differentially affected by gestational age and PIH. Special attention was given to differentially regulated pathways associated with *MRPL38* and *FKBP8* interaction genes because these had additional functional enrichments beyond mitochondrial function and homeostasis. Many of these pathways (69.5%) have been implicated in PIH. The broad pattern of differentially regulated pathways carried out by *MRPL38* and *FKBP8* interaction genes showed cell death/survival pathways as downregulated in early pregnancy with a transition to upregulation in late pregnancy, and immune system/inflammatory pathways as upregulated in early pregnancy with either attenuation in late pregnancy or a transition to downregulation. This opposing pattern may be a mitigation response to overactivation of inflammatory processes in early gestation. Although very early pregnancy is normally an inflammatory state as the allogenic placenta becomes established within the uterus (52), immunologic imbalance favoring excess inflammation during this time is often associated with pathologic states such as preeclampsia (53).

Several *MRPL38* and *FKBP8* interaction gene pathways participate in vascularization of the placenta and act on the vascular endothelium independent of pregnancy (e.g., EIF2 signaling, Sirtuin signaling, VEGF signaling, and eNOS signaling). Differential regulation of these pathways respective to normotensive pregnancies supports the angiogenic imbalance hypothesis (54), where dysregulated angiogenesis plays a major pathogenic role in PIH. It additionally suggests mitochondrial function and/or efficiency is a mediating factor in angiogenic imbalance. Peroxisome proliferator-activated receptor (PPAR) signaling was also differentially regulated in PIH pregnancies compared with normotensive pregnancies. The PPAR protein family, most notably PPARγ, are vital for placental development in normal pregnancy. In humans, PPARγ is highly expressed in trophoblasts and is crucial for both trophoblast differentiation and normal development of the placental vasculature (55). The transition to upregulated PPAR signaling in later gestation in PIH cases observed here may reflect pathway cross talk or compensatory processes that ensure offspring survival, as PPARγ is expected to be consistently lower in serum from mothers with preeclampsia (56, 57).

Limited mtDEG interaction genes were differentially expressed at delivery in both the maternal and fetal data sets. This suggests that mitochondria-mediated biological processes at delivery are not associated with PIH pathophysiology, or that mitochondria-mediated processes associated with PIH are alleviated at delivery. Functional enrichment of mtDEG interaction genes at delivery showed a single biological process term (vesicles) was shared between maternal and fetal data sets. Although this could be an artifact of sample tissue origin, the preparation for labor and delivery at the end of pregnancy may also drive the different processes observed here.

Fetal mtDEG interaction genes displayed a prominent signal for release of EVs in PIH. As placenta-derived EVs are more abundant in pregnancies with preeclampsia (58), this finding suggests altered placental mitochondrial function may in turn alter cell-cell communication, possibly via delivery of placental factors into maternal circulation that can affect maternal physiology. Our previous work demonstrated that circulating cell-free mtDNA in late pregnancy primarily exist in a membrane-bound state (8). Others have further shown the presence of sFLT1 in placenta-derived EVs (59, 60). Though *FLT1* is not an mtDEG interaction gene, it was upregulated at delivery in the PIH placentas examined here (17). If this is true throughout pregnancy, it may specifically implicate fetal inheritance of mitochondrial dynamics as an important contributor to PIH, particularly as it pertains to placental function and communication between mother and fetus in pregnancies with de novo hypertension.

The maternal mitochondrial genome derived from PBMCs did not display differential mutational loads between normotensive pregnancies and pregnancies with preeclampsia. This finding was unexpected given the abundance of evidence for mitochondrial involvement in preeclampsia pathophysiology (8, 61–64). In addition, mtDNA harboring oxidative lesions is more immunogenic (65–68) and is also directly linked to mutagenesis/mutational load (68–70). Increased mutations may further contribute to inflammatory pathway activation particularly because activated maternal mononuclear cells are more abundant during pregnancy due to roles in placentation (71, 72). It is possible that this is an artifact of small sample size. The medium effect size in mutational load, however, indicates biological significance that may reach statistical significance if appropriately powered. Alternatively, other mechanisms, such as epigenetic modification, may instead drive the disconnect between the presence of differential mitochondrial gene expression and lack of changes in the mitochondrial genome mutational load between PIH and normotensive subjects.

### Study Limitations, Strengths, and Perspectives

In this study, we have made the assumption that the dominant and primary signal in the maternal circulating cell-free transcriptome is maternally derived. During pregnancy, placental DNA and RNA are shed and enter the maternal circulation, though the levels of placental RNA are relatively low, with <1% of the maternal circulating cell-free transcriptome at the end of the 1st trimester deriving from the placenta, which increases to <4% in the second trimester, and peaks at <16% in the 3rd trimester (14). However, we do not distinguish the origin of each differentially expressed transcript and therefore cannot say with certainty that the majority of significant differentially expressed genes in our maternal data set are maternally derived.

There are some limitations resulting from the use of data generated from previous studies. Some important subject characteristics (e.g., medications administered to mothers, therapies or treatments mothers underwent) were not disclosed in these data, which may have introduced confounding variables that could not be accounted for. In addition, because our study sample sizes were lower than ideal, the differences between PIH and normotensive subjects required larger effect sizes for detection, and other significant trends with smaller effect sizes may have been missed. Further, the maternal transcriptomic data that were used pooled together patients with preeclampsia and gestational hypertension to define PIH. It should be noted that *1*) both gestational hypertension and preeclampsia are considered acute (de novo) hypertensive disorders of pregnancy (as compared with chronic hypertension); *2*) gestational hypertension may be considered subclinical preeclampsia (73); *3*) the American College of Obstetrics and Gynecology has recommended that patients with gestational hypertension presenting with severe-range blood pressures should be managed with the same approach as those with severe preeclampsia (1). Nevertheless, we acknowledge that gestational hypertension, preeclampsia, and its subtypes may differ in etiology. Though we stress that the objective of this study was to investigate the presence of a shared mechanism underlying the pathology of these disorders (rather than causative factors), pooling hypertensive subjects may have obscured biologically meaningful mitochondrial signals that may have otherwise been detected. The use of different study populations between maternal and fetal data sets may have also hindered our ability to conclude if similar mtDEGs or mitochondrial processes are dysregulated in both PIH mothers and fetuses. This can be driven by factors such as using different Illumina HiSeq platforms or differences in analytical approaches. Future work would benefit from pairing placental transcriptomics with prospective longitudinal monitoring of the cell-free transcriptome within the same mothers.

An additional limitation is that functional assays to confirm specific mitochondrial involvement in PIH pathophysiology were not performed, as they were above and beyond the scope of this study. As a result, we cannot say with certainty that the implicated genes and pathways are truly dysregulated. Protein levels often do not correlate well to transcript levels, and posttranscriptional or posttranslational mechanisms can further influence protein levels and isoforms. However, there is evidence that significantly differentially expressed transcripts do display significantly higher correlation coefficients with protein levels (74). This work, therefore, presents a platform to guide future research, as altered mitochondria-mediated pathways appear to be common in de novo hypertensive disorders of pregnancy.

**Figure 5.**
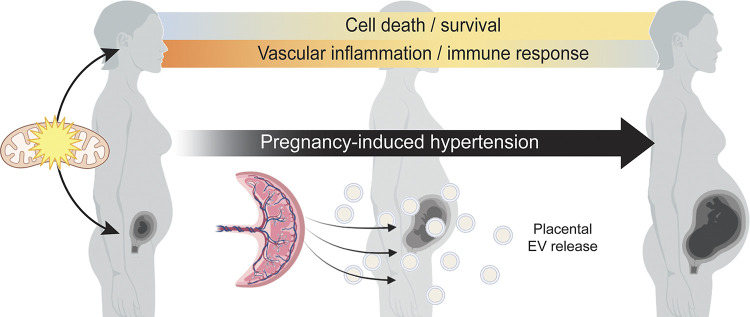
Hypothetical model depicting two-pronged effects of mitochondrial dysregulation in pregnancy-induced hypertension development. In mothers with pregnancy-induced hypertension, mitochondrial dysregulation leads to differential regulation of cell death/survival pathways and inflammatory/immune response pathways. In the developing fetus, inheritance of mitochondrial dysregulation affects maternal-fetal communication through increased extracellular vesicle (EV) release from the placenta. [Image created with BioRender.com and published with permission.]

In conclusion, our findings expand knowledge about the role of mitochondria in the pathophysiology of acute hypertensive disorders of pregnancy, namely preeclampsia and gestational hypertension. Longitudinal analysis of systemic expression of mtDEG interaction genes via the maternal circulating cell-free transcriptome demonstrated differential regulation of pathways implicated in PIH as early as the first trimester. Our data connect pregnancy-specific mitochondrial dysregulation with established preeclampsia-associated processes and inflammation. Though this may only be specific to delivery, increased production, and release of placental EVs during pregnancy may also be mediated by mitochondria. Figure 5 depicts a proposed theoretical model, in which maternal mitochondrial dysregulation contributes to an over-inflammatory state that is further promoted by placental release of EVs into the maternal circulation. Converging or common mitochondria-mediated pathways underlying development of gestational hypertension and preeclampsia warrant further investigation.

## DATA AVAILABILITY

All analysis code and working files are openly available at Github (https://github.com/contessaricci/mito_dysfunction_PIH). Additional data are available from the corresponding authors upon reasonable request.

## SUPPLEMENTAL DATA

10.6084/m9.figshare.21970706 Supplemental Figs. S1–S5: https://doi.org/10.6084/m9.figshare.21970706.

10.6084/m9.figshare.21970694 Supplemental Tables S1–S5: https://doi.org/10.6084/m9.figshare.21970694.

10.6084/m9.figshare.21970595 Supplemental File S1: https://doi.org/10.6084/m9.figshare.21970595.

10.6084/m9.figshare.21970655 Supplemental File S2: https://doi.org/10.6084/m9.figshare.21970655.

10.6084/m9.figshare.21970664 Supplemental File S3: https://doi.org/10.6084/m9.figshare.21970664.

10.6084/m9.figshare.21970667 Supplemental File S4: https://doi.org/10.6084/m9.figshare.21970667.

10.6084/m9.figshare.21970670 Supplemental File S5: https://doi.org/10.6084/m9.figshare.21970670.

## GRANTS

This research was supported in part by the National Institutes of Health (R01HL0146562; R01HL146562-04S1; R01HD089940), National Institutes on Minority Health and Health Disparities of the National Institutes of Health (U54MD006882), American Heart Association (18SCG34350001, 15SFRN 23730000, 15SFRN 23480000), the University of Iowa Carver College of Medicine, and the National Center for Advancing Translational Sciences of the National Institutes of Health (UL1TR002537, UL1TR002537-S1).

## DISCLAIMERS

The content is solely the responsibility of the authors and does not necessarily represent the official views of funders.

## DISCLOSURES

D. A. Santillan and M. K. Santillan hold patents related to the prediction and treatment of preeclampsia: US 217 293 No. 9,937,182 (April 10, 2018), EU No. 2,954,324, and PCT/US2018/027152. None of the other authors has any conflicts of interest, financial or otherwise, to disclose. 

## AUTHOR CONTRIBUTIONS

C.A.R., N.R.P., and S.G. conceived and designed research; C.A.R., D.M.R., J.S., D.A.S., M.K.S., and N.R.P. performed experiments; C.A.R., D.M.R., J.S., N.R.P., and S.G. analyzed data; C.A.R., D.M.R., D.A.S., M.K.S., N.R.P., and S.G. interpreted results of experiments; C.A.R., N.R.P., and S.G. prepared figures; C.A.R., N.R.P., and S.G. drafted manuscript; C.A.R., D.M.R., D.A.S., M.K.S., N.R.P., and S.G. edited and revised manuscript; C.A.R., D.M.R., J.S., D.A.S., M.K.S., N.R.P., and S.G. approved final version of manuscript.
